# Intramolecular Epistasis and the Evolution of a New Enzymatic Function

**DOI:** 10.1371/journal.pone.0039822

**Published:** 2012-06-29

**Authors:** Sajid Noor, Matthew C. Taylor, Robyn J. Russell, Lars S. Jermiin, Colin J. Jackson, John G. Oakeshott, Colin Scott

**Affiliations:** 1 Ecosystem Sciences, Commonwealth Scientific and Industrial Research Organisation, Canberra, Australian Capital Territory, Australia; 2 School of Biological Sciences, University of Sydney, Sydney, New South Wales, Australia; 3 Department of Biochemical Sciences “Rossi Fanelli,” University of Rome “La Sapienza,” Rome, Italy; 4 Research School of Chemistry, Australian National University, Canberra, Australian Capital Territory, Australia; University of Canterbury, New Zealand

## Abstract

Atrazine chlorohydrolase (AtzA) and its close relative melamine deaminase (TriA) differ by just nine amino acid substitutions but have distinct catalytic activities. Together, they offer an informative model system to study the molecular processes that underpin the emergence of new enzymatic function. Here we have constructed the potential evolutionary trajectories between AtzA and TriA, and characterized the catalytic activities and biophysical properties of the intermediates along those trajectories. The order in which the nine amino acid substitutions that separate the enzymes could be introduced to either enzyme, while maintaining significant catalytic activity, was dictated by epistatic interactions, principally between three amino acids within the active site: namely, S331C, N328D and F84L. The mechanistic basis for the epistatic relationships is consistent with a model for the catalytic mechanisms in which protonation is required for hydrolysis of melamine, but not atrazine.

## Introduction

The evolutionary mechanisms by which new catalytic functions of enzymes emerge have attracted considerable attention in recent times. Advances in our understanding of these processes have been greatly accelerated by developments in laboratory-based evolution of enzymes [1], [2]. Such studies have highlighted the importance of enzymatic promiscuity [3] and trade-offs between the emergent activity and the catalytic and non-catalytic properties (e.g., stability) of the parent enzyme [4]. Despite these advances, there have been few studies of natural systems in which evolution of a new function has been characterized at a molecular level [5], [6], [7]. An opportunity to study the process that underpins the emergence of new enzymatic activities in natural systems is presented by bacterial enzymes that have recently diverged from their ‘natural’ physiological functions to acquire potentially useful roles in xenobiotic degradation [8], [9], [10].

**Figure 1:**
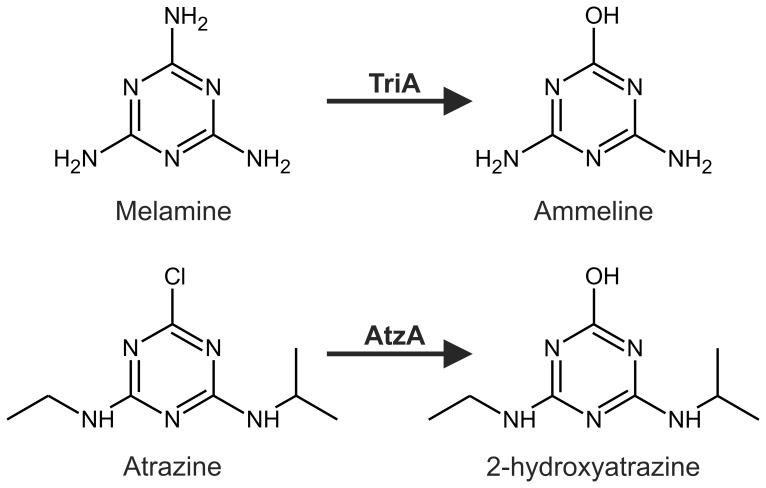
Reaction schemes for melamine deaminase (TriA) and atrazine dechlorinase (AtzA). The hydrolytic deamination of melamine to ammeline by TriA and the dechlorination of atrazine to 2-hydroxyatrazine by AtzA are shown. TriA also possesses a low level of atrazine dechlorinase activity [17].

Atrazine dechlorinase (AtzA) and its close relative melamine deaminase (TriA) offer an excellent model system to study the evolution of new enzyme function. AtzA and TriA were first described in two different *Pseudomonas* species; AtzA from atrazine-contaminated soil (*Pseudomonas* sp. strain ADP1 [11], [12]) and TriA from effluent from a melamine manufacturing plant (*Pseudomonas* sp. strain NRRLB-12227 [13]). These two bacterial species appear to be well adapted for the use of the respective xenobiotics as nitrogen and carbon sources. *Pseudomonas* sp. strain ADP1 is capable of mineralizing atrazine and its metabolites (but not melamine) and *Pseudomonas* sp. strain NRRLB-12227 is capable of mineralizing melamine and its metabolites (but not atrazine) [13], [14]. The difference in metabolic capability is partly explained by differences in specificity between AtzA and TriA; AtzA is an efficient atrazine dechlorinase with no measurable deaminase activity, and TriA is an efficient deaminase with only a low level of promiscuous dechlorinase activity (Fig. 1) [15], [16], [17].

**Figure 2:**
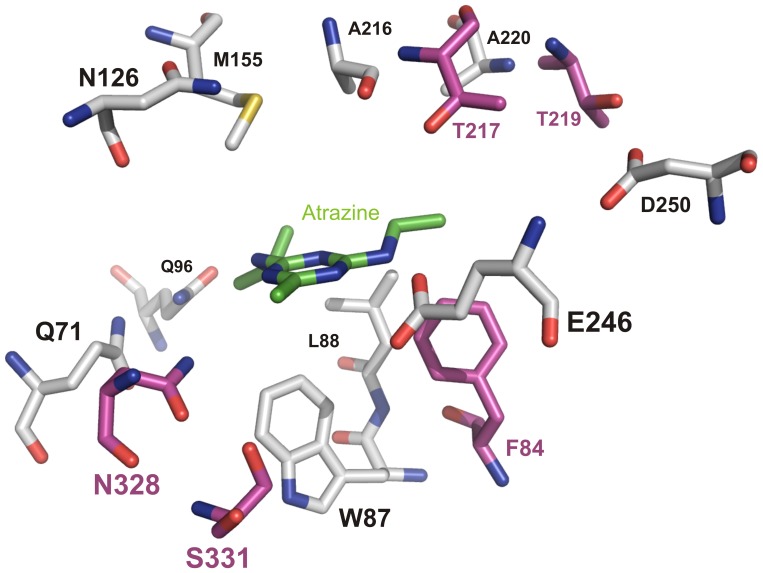
Modeled active site of AtzA. A homology model of the active site of AtzA [20] was used to illustrate the positions of five of the nine amino acid differences between AtzA and TriA. Shown here are the AtzA substrate (atrazine; green), amino acids identical in both AtzA and TriA (Q71, W87, L88, Q96, N126, M155, A216, A220, E246 and D250; white), and amino acids that differ between AtzA and TriA (positions 84, 217, 219, 328 and 331; purple).

AtzA and TriA are metal-dependent hydrolases with 98% sequence identity to each other, differing by just 9/475 amino acids [i.e., F84L, V92L, E125D, T217I, T219P, I253L, G255W, N328D and S331C, with respect to the AtzA sequence: 17]. At least five of these substitutions are located within the active site of the enzyme (Fig. 2), and there are no synonymous differences between the two genes [17]. It is unclear which of AtzA or TriA most closely resembles the ancestral *vs.* derived condition, but it is assumed that their divergence has been recent [17].

AtzA has now been isolated from geographically and phylogenetically diverse bacterial species [18]. Notably, however, little sequence variation has been found between the *atzA* gene from *Pseudomonas* sp. strain ADP1 and those isolated later. There have also been no reports of *triA* and *atzA* sequences being obtained from the same organism, or even from the same location.

Raillard et al. [16] randomly recombined the genes encoding AtzA and TriA by DNA shuffling and screened over 400 variants for activity against melamine, atrazine, and a range of related triazine compounds. A high degree of plasticity in substrate usage was observed in several intermediates between the two enzymes. This suggests that there may not have been a strong negative trade-off of the original activity regardless of the direction in which evolution proceeded between AtzA and TriA (or related enzymes).

Step-wise transitions, mimicking the possible paths of natural selection, between closely related naturally occurring enzyme variants have been constructed elsewhere [5], [6], [19]. However, a step-wise transition has not previously been explored for AtzA and TriA. Here, we have constructed potential evolutionary trajectories between AtzA and TriA and used these trajectories to examine the reversibility of evolution between these enzymes, the trade-offs in their catalytic and biophysical properties, and the constraints upon the order of amino acid substitutions enforced by intramolecular epistasis.

## Results

### Constructing a Step-wise Trajectory from AtzA to TriA

**Figure 3:**
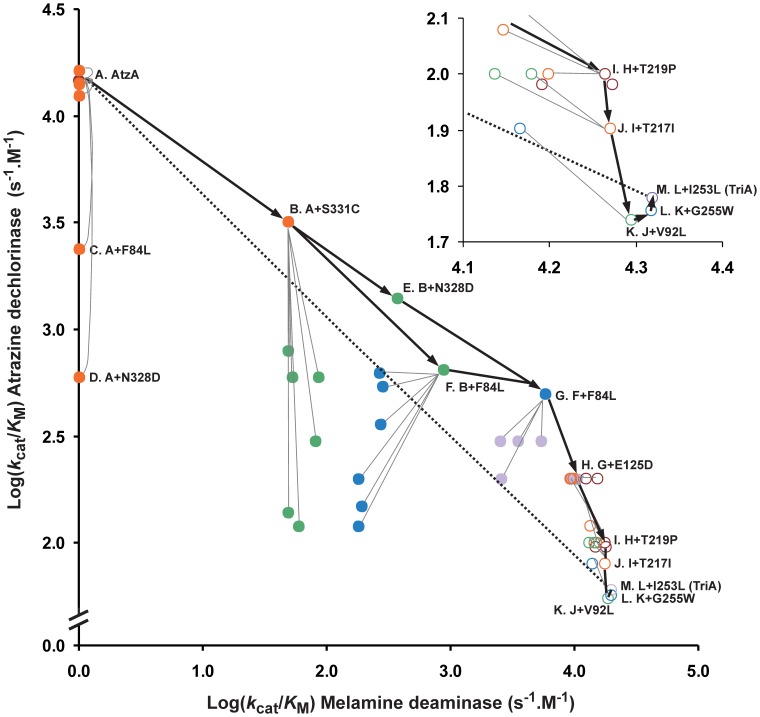
Step-wise laboratory-based evolution of AtzA to TriA. Circles indicate the variants for which the *k*_cat_/*K*_M_ values (s^−1^.M^−1^; values in Table 1 and Table S2) for atrazine dechlorination and melamine deamination were determined (color coded as follows: AtzA, red (filled); generation 1, orange (filled); generation 2, green (filled); generation 3, blue (filled); generation 4, violet (filled); generation 5, orange (open); generation 6, red (open); generation 7, green (open); generation 8, blue (open); and TriA, violet (open)). Lines are used to link variants differing by one substitution (thick lines link optimal variants; thin lines link the optimal variants to suboptimal variants – suboptimal variants were not used to generate subsequent variants). Amino acid substitutions discussed in the text have been labelled for clarity, as have the wild-type AtzA and TriA enzymes. Inset: expansion of the region of Fig. 3 that contains the last four steps of the trajectory.

AtzA was used as the template for the sequential introduction of the nine amino acid substitutions that separate it from TriA. As previously reported [12], [20], AtzA had no detectable deaminase activity with melamine and a *k*_cat_/*K*_M_ value of 14,600 s^−1^.M^−1^ for atrazine (Table 1). After each round of mutagenesis, the variant enzyme with the greatest gain in melamine deaminase specificity (*k*_cat_/*K*_M_) was used as the template for the next round of these experiments. Data for the enzyme purifications are given in Fig. S1, and kinetic data for the variants are in Table 1 and Table S2. The specificities for each enzyme variant for both substrates are plotted graphically in Fig. 3.

**Table 1:** Second order rate constants for atrazine, melamine and ametryn for the AtzA and TriA variants that comprise the major trajectories between AtzA and TriA and *vice versa*.

		*k*_cat_/*K*_M_ (s^−1^.M^−1^)		
Direction and Generation	Variant	Atrazine (-Cl)	Melamine (-NH_2_)	Ametryn (-OCH_3_)
AtzATriA				
0	A. AtzA	14,600±634	BDL	BDL
1	B. A plus S331C	3,200±222	50±4	127±9
1	C. A plus F84L	2,400±128	BDL	ND
1	D. A plus N328D	600±3	BDL	ND
2	E. B plus N328D	1,400±135	380±28	395±12
2	F. B plus F84L	650±63	910±79	ND
3	G. F plus N328D	512±43	6,100±573	411±27
4	H. G plus E125D	213±14	10,898±890	422±25
5	I. H plus T219P	96±7	18,701±1,170	392±30
6	J. I plus T217I	80±7	18,604±1,386	404±22
7	K. J plus V92L	55±6	19,700±1,670	382±28
8	L. K plus G255W	57±5	20,760±1,677	437±33
9	M. L plus I253L (TriA)	60±6	20,810±1,581	425±33
TriA <$>\scale 70%\raster="rg2"<$>AtzA				
0	M. TriA	60±6	20,810±1,581	425±33
1	N. M plus C331S	1,689±144	3,298±277	ND
1	O. M plus D328N	BDL	BDL	BDL
1	P. M plus L84F	821±68	6,103±821	BDL
2	Q. P plus D328N	BDL	BDL	BDL
2	R. N plus L84F	3,803±275	1,107±99	ND
2	S. N plus D328N	9,812±484	0	ND
3	T. S plus L84F	12,300±879	0	ND

The *k*_cat_/*K*_M_ values (± standard deviation) are shown. The *k*_cat_/*K*_M_ values for the all of the variants shown in Fig. 2 and Fig. 3 can be found in Table S2. The leaving group for each hydrolysis is shown in parentheses next to the name of the substrate. ND  =  Not determined; BDL  =  below detection limit. Each variant has been assigned a letter and the identity of the each variant’s direct parent is indicated together with the distinguishing amino acid substitution. The letter assignments correspond to those found in the figures.

Among the nine first-step variants, only the S331C substitution conferred an increase in melamine deaminase activity, with a *k*_cat_/*K*_M_ value of 50 s^−1^.M^−1^ (Table 1; Fig. 3). This substitution resulted in a 4.6-fold trade-off in atrazine dechlorinase specificity (3,200 s^−1^.M^−1^). Despite not increasing the deaminase activity, the F84L and N328D variants led to 6.1-fold (2,400 s^−1^.M^−1^) and 24.3-fold (600 s^−1^.M^−1^) reductions in atrazine dechlorinase specificities, respectively. The remaining six first-step variants (V92L, E125D, T217I, T219P, I253L, and G255W) had no significant effect on either activity (Fig. 3; Table S2).

The gene encoding the S331C variant of AtzA from generation 1 was then used as a template for the introduction of the other eight amino acid substitutions. The *k*_cat_/*K*_M_ values for melamine increased 18.2-fold and 7.6-fold following the F84L (910 s^−1^.M^−1^) and N328D (380 s^−1^.M^−1^) substitutions, respectively (Fig. 3), whereas no substantial increase was observed for the other six variants (Fig. 3; Table S2). All eight of the second-step variants significantly reduced atrazine dechlorinase activities compared with the S331C variant of AtzA, with *k*_cat_/*K*_M_ values of 4.9- and 2.3-fold decreases for F84L (650 s^−1^.M^−1^) and N328D (1,400 s^−1^.M^−1^) substitutions, respectively. There was a positive epistatic interaction between the S331C substitution and the F84L and N328D substitutions, as neither of the latter two substitutions had increased the enzyme’s catalytic activity in relation to atrazine in the AtzA wild-type background (Fig. 3; Table 1; Table S2).

In the next step (generation 3), the S331C-F84L variant of AtzA (Table 1) was used as template for the introduction of the other seven amino acid substitutions, with the only significant increase in melamine deaminase activity resulting from the N328D substitution. This S331C-F84L-N328D variant had a 6.7-fold and 16-fold higher deaminase activity than the S331C-F84L and S331C-N328D variants, respectively. These increases in deaminase activity resulted in 4.9-fold and 2.3-fold reductions in the dechlorinase activity. Because the effects of F84L and N328D substitutions were additive in the S331C background, it appears that there is no epistatic interaction between them.

The optimal order of the remaining amino acid substitutions was E125D <$>\scale 70%\raster="rg2"<$> T219P <$>\scale 70%\raster="rg2"<$> T217I <$>\scale 70%\raster="rg2"<$> V92L <$>\scale 70%\raster="rg2"<$> G255W <$>\scale 70%\raster="rg2"<$> I253L. Each substitution had a relatively small effect on the melamine deaminase activity but in combination served to greatly reduce the dechlorinase activity (Fig. 3; Table 1; Table S2).

### Construction of a Step-wise Trajectory from TriA to AtzA

**Figure 4:**
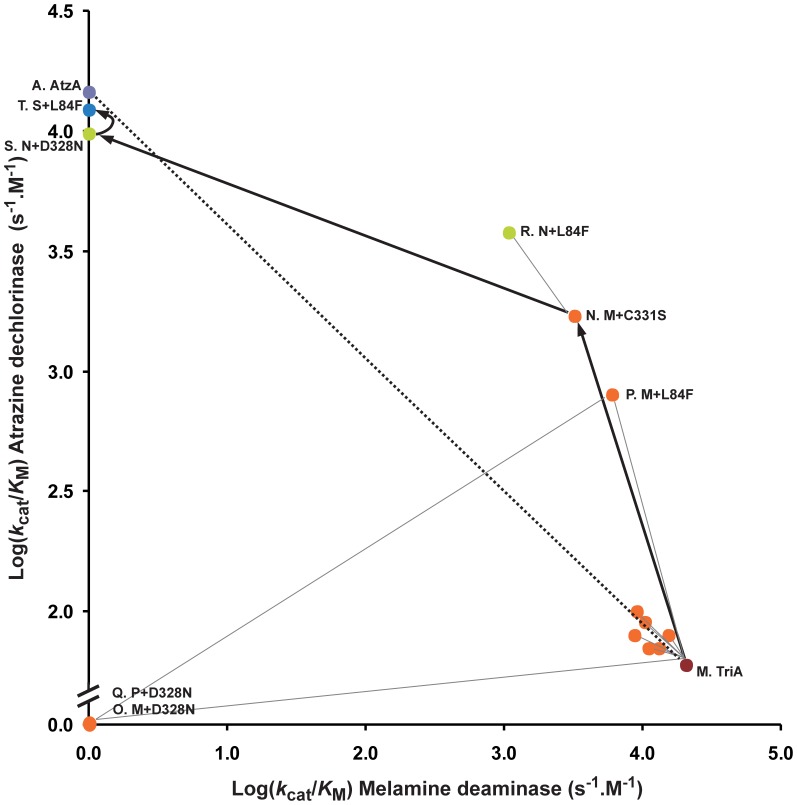
Partial step-wise laboratory evolution of TriA to AtzA. Circles indicate the variants for which the *k*_cat_/*K*_M_ values (s^−1^.M^−1^; values in Table 1 and Table S2) for atrazine dechlorination and melamine deamination were determined, and are color coded as follows: TriA, red; generation 1, orange; generation 2, green; generation 3, blue; AtzA, violet. Lines are used to link variants differing by one substitution (thick lines link optimal variants; thin lines link the optimal variants to suboptimal variants – suboptimal variants were not used to generate subsequent variants). Amino acid substitutions discussed in the text have been labelled for clarity, as have the wild-type AtzA and TriA enzymes.

The evolutionary trajectory in the direction from TriA to AtzA was also constructed (Table 1; Fig. 4; Table S2). The strategy for this experiment was the same as above, except that, in this case, the selection criterion at each step was the highest level of atrazine dechlorinase activity.

TriA already exhibited low-level atrazine dechlorinase activity and the addition of a C331S mutation in the first generation gave by far the greatest increase in specificity (24.3-fold; i.e., from 70 s^−1^.M^−1^ to 1,700 s^−1^.M^−1^), with a smaller increase observed as a result of the L84F substitution (11.4-fold). Six of the other amino acid substitutions yielded no significant increase in specificity towards atrazine (1.0-1.4 fold for L92V, D125E, I217T, P219T, L253I and W255G) whereas the seventh amino acid substitution (D328N) resulted in the loss of all catalytic activity. Given that the D328N mutant was soluble (Fig. S1), the loss of detectable activity must result from effects at the active site, where D328 is predicted to be located.

Notably, the D328N substitution caused an increase in atrazine dechlorinase activity, with a 5.8-fold increase in specificity constant when added to the C331S variant (i.e., 9,800 s^−1^.M^−1^ or 67% of wild-type AtzA activity). This C331S-D328N variant possessed no melamine deaminase activity. On the other hand, when the D328N substitution was added to the L84F variant of TriA (made in the following round), it inactivated the enzyme completely (Table 1; Fig. 4). Interestingly, the C331S-D328N-L84F variant of TriA was active and had a higher atrazine dechlorinase activity (*k*_cat_/*K*_M_ value of 12,300 s^−1^.M^−1^) than that of the C331S- D328N variant of TriA. The atrazine dechlorinase activity of this triple variant was 84% of that of the wild-type AtzA. The other six amino acid substitutions increased the catalytic activity towards atrazine marginally (Fig. 4 and data not shown) although in combination they bridged the gap between the catalytic activity of the triple variant and that of the wild-type AtzA.

### Trade-offs between Physiological and Promiscuous Activities

Raillard et al. [16] noted significant promiscuity in TriA (hydrolysis of -SCH_3_ and -OCH_3_ moieties from triazine rings) that was not present in AtzA. We have also observed that TriA can hydrolyse –SH and –CF_3_ groups from the 2-position of triazine rings, whilst AtzA cannot (Table S2).

**Figure 5:**
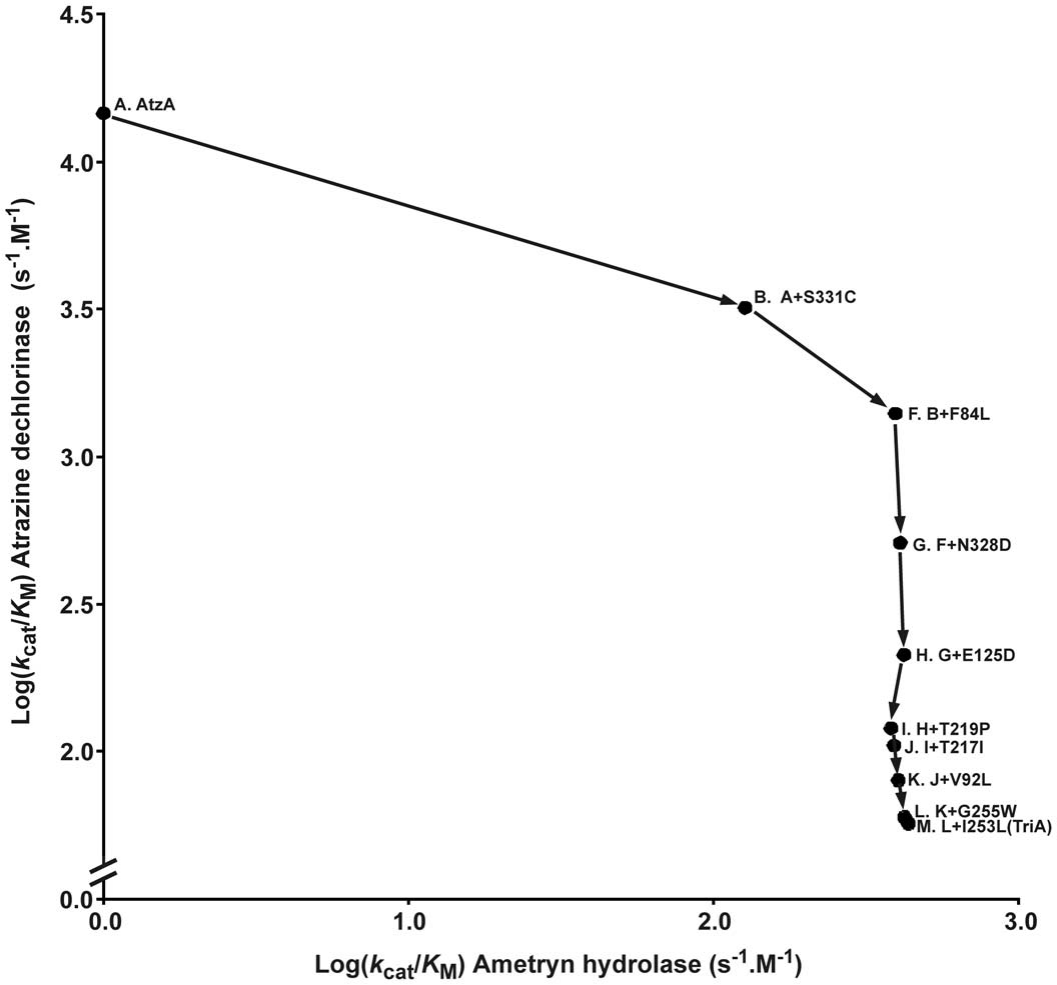
Trade-off between atrazine dechlorinase and ametryn hydrolase activity during the transition between AtzA and TriA. Circles indicate the variants for which the *k*_cat_/*K*_M_ values (s^−1^.M^−1^; values in Table 1 and Table S2) for atrazine dechlorination and ametryn hydrolysis are shown. Lines are used to link variants differing by a single amino acid. Each variant has been assigned a letter and the identity of the each variant’s direct parent is indicated together with the distinguishing amino acid substitution. The letter assignments correspond to those found in Fig. 3 and Table 1.

To study how physiological and non-physiological activities trade-off with each other, the specificities of the variants along the trajectory from AtzA to TriA for ametryn (N-ethyl-N′-(1-methylethyl)-6-(methylthio)-1,3,5-triazine-2,4-diamine; possessing a –SCH_3_ leaving group) hydrolysis were therefore also assessed (Fig. 5). The majority of the increase in ametryn hydrolase specificity was accounted for by the introduction of the S331C and N328D substitutions, with little contribution by the remaining substitutions. S331C and N328D had also been the most influential substitutions in terms of the acquisition of melamine deaminase activity in the AtzA-TriA transition.

### Activity vs. Stability Trade-off

**Figure 6:**
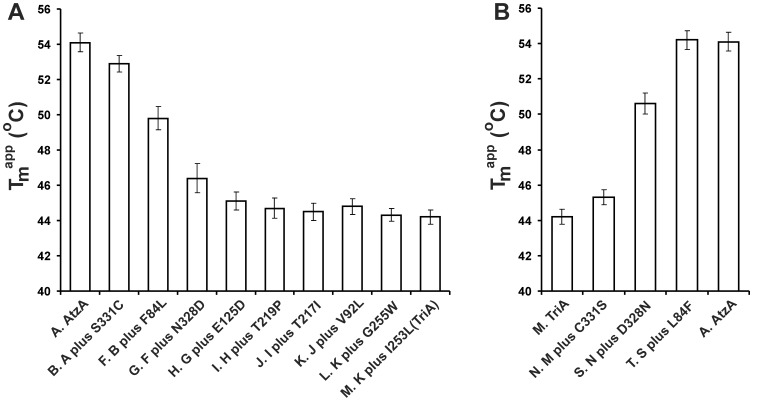
Apparent melting temperatures (T_m_^app^) of AtzA, TriA and their intermediates. T_m_^app^ of the enzyme variants along the step-wise trajectories from AtzA to TriA (A) and from TriA to AtzA (B) calculated from residual enzyme activities after heating for 15 minutes at temperatures between 30°C and 70°C. Each variant has been assigned a letter and the identity of the each variant’s direct parent is indicated together with the distinguishing amino acid substitution. The letter assignments correspond to those found in Figs. 3 and 4 and Table 1. Error bars indicate 95% confidence limits.

Conformational stability is frequently observed to trade-off with activity during the evolution of new enzyme function [4], [21], [22]. To investigate the effects of the nine substitutions on stability, the stabilities of AtzA, TriA, and the intermediates found along the two trajectories in Figures 3 and 4 were therefore assessed using residual catalytic activity after heat treatment (30–70°C for 15 minutes) (Fig. 6A).

For the trajectory from AtzA to TriA, the thermal stability (T_m_^app^) of AtzA began at 54°C and the successive addition of the S331C, F84L, N328D, and E125D substitutions successively reduced the T_m_^app^ of the enzyme, to a final value of 44°C (Fig. 5A). Conversely, the first three steps in the direction from TriA to AtzA (C331S, L84F, and D328N) increased the thermal stability of the enzyme, with the T_m_^app^ increasing from approximately 44°C for TriA to approximately 54°C for the C331S-D328N-L84F variant of TriA (Fig. 6B).

## Discussion

### Intramolecular Epistasis Constrains the Evolutionary Trajectories

It is becoming clear that intra- and inter-molecular epistasis may contribute significantly to the availability of evolutionary trajectories by constraining the order and reversibility of amino acid substitutions [5], [6], [7], [23], [24].

**Figure 7:**
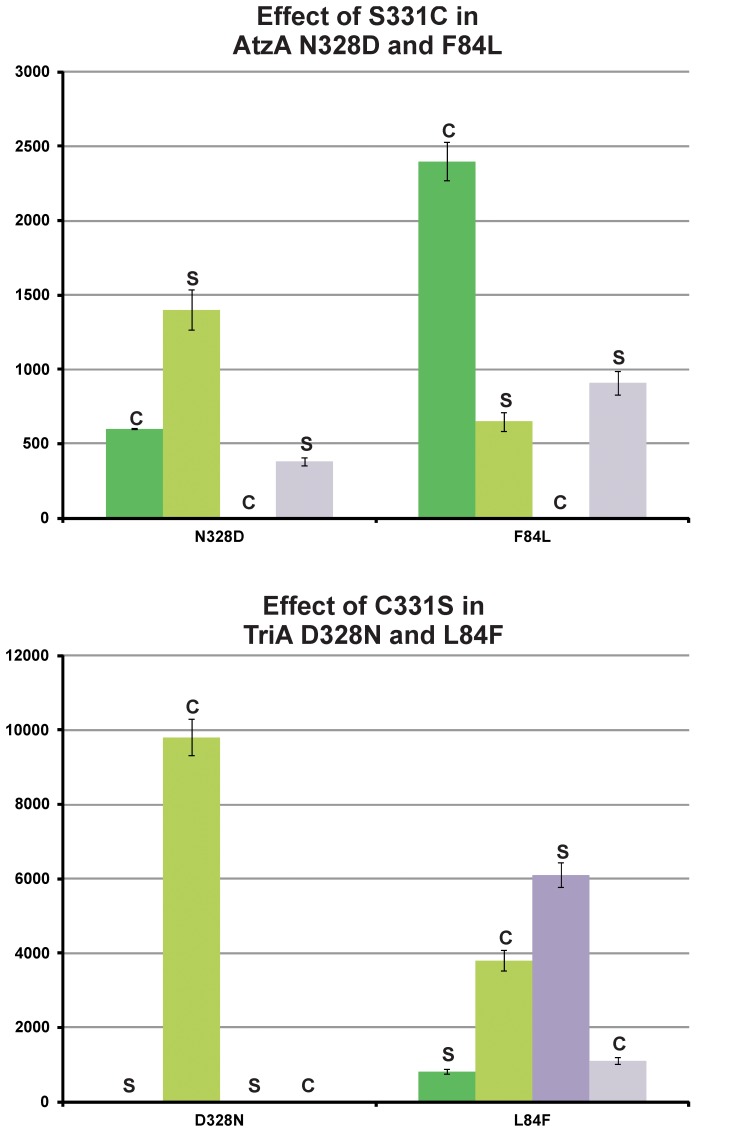
Epistatic effects of the C331S substitution in AtzA and the S331C substitution in TriA. The *k*_cat_/*K*_M_ values for atrazine dechlorination (green) and melamine deamination (blue) in the wild-type (dark) or position 331 variant (light) are shown for the substitutions at positions 328 and 84 in AtzA (top) and TriA (bottom). The identity of the amino acid at position 331 (cysteine, C, or serine, S) is indicated for clarity. Error bars indicate standard deviations.

The evolution between AtzA and TriA is reversible; however the preferred orders of events along the two trajectories are not simply the reverse of one another, owing to strong epistatic effects between the substitutions at residues 331, 328, and 84 (Fig. 7). The interaction between amino acids at positions 328 and 331 entails that substitutions at position 331 must precede those at position 328 in either direction (Fig. 7). Substitutions at position 328 that precede those at position 331 are deleterious, either reducing (in the AtzA to TriA direction) or abolishing (in the TriA to AtzA direction) parental activity, with no enhancement of the alternative activity. The evolution of TriA from AtzA also requires that the F84L substitution be preceded by the S331C substitution, although this constraint is relaxed in the trajectory from TriA to AtzA. Although the trajectories constructed here are only potential trajectories, and others are plausible, it is very likely that the order of the substitutions at positions 84, 328 and 331 are as described here due to their epistatic interactions.

**Figure 8:**
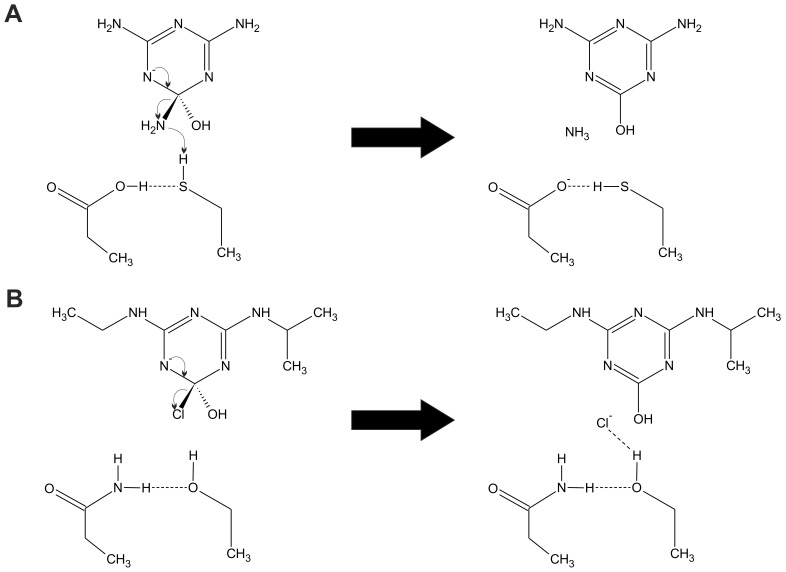
Roles of Amino Acids at Positions 328 and 331 in AtzA and TriA. In TriA (A) Cys331 donates a proton to the NH_2_− leaving group of melamine and abstracts a proton from Asp328. In AtzA (B), the serine hydroxyl group stabilizes the halide of atrazine in the transition state via a hydrogen bonding interaction and is in turn stabilized by Asn328.

The molecular basis for these epistatic interactions can be rationalized using the catalytic models for AtzA and TriA that we have proposed elsewhere [20] (Fig. 8). Different requirements for leaving group stabilization in AtzA and TriA give rise to the epistatic interactions between the residues at positions 328 and 331. With a p*K*_a_ value of -7 for HCl/Cl^−^, the Cl^−^ leaving group of atrazine will be stable in this form, whereas the melamine leaving group NH_3_/NH_2_^−^, with a p*K*_a_ value of 34, will require protonation from an NH_2_^−^ to NH_3_ group for catalysis to be efficient. Thus, for the TriA reaction, cysteine most likely acts as a proton donor, a role that cannot be fulfilled by serine. The second member of the dyad then serves to stabilize the first: in the case of TriA, C331 can abstract a proton from Asp328 as it donates a proton to the leaving group of melamine, preventing formation of a high-energy thiolate.

The epistasis between the two members of the dyad can then be broken down as follows: for the deaminase activity, if the residue at position 331 is serine, an N328D mutation has no effect on the activity since S331 cannot donate a proton to the leaving group regardless of the other member of the dyad. In contrast, if the N328D mutation arises after the C331 mutation, it has a positive effect because, unlike asparagine, it is able to shuttle a proton to C331 as C331 donates its proton to the leaving group. Thus, the intramolecular epistasis observed in this evolutionary trajectory can be traced back to the reaction chemistry.

### Functional Trade-offs and Evolution

As expected, there is a clear trade-off between the two enzymatic activities during the transition from AtzA to TriA or *vice versa*. The pair-wise plot of the two activities in the AtzA to TriA direction shows a profile in which the intermediate variants possess both new and native activities, with the native activity lost gradually while the new activity is established (Fig. 3). This profile is strikingly similar to the theoretical model of a weak-negative trade-off proposed by Khersonsky and Tawfik. [3]. However, the pair-wise plot of the trajectory from TriA to AtzA (Fig. 4) shows that there is large trade-off in activities along this trajectory, with TriA activity totally abolished with the addition of the second amino acid substitution (a strong negative trade off).

There is also a trade-off between enzymatic activity and protein stability. However, this trade-off only operates in the AtzA to TriA direction. The substitutions that confer the greatest increases in TriA activity along this trajectory (S331C, D328N and F84L) also have the greatest destabilizing effect on the thermal stability of the enzyme. Conversely, introduction of substitutions at these positions in the TriA background substantially improves thermal stability.

*In vitro* evolution studies have provided a considerable body of evidence showing that destabilizing effects almost always accompany mutations that confer new catalytic functions upon enzymes [4], [21], which could suggest that the dechlorinase function is ancestral in this case. However, we cannot support this conjecture with a phylogenetic analysis as there are too few informative sequences available to construct a phylogeny with sufficient resolution to identify which activity (deaminase or dechlorinase) has diverged most recently.

Additionally, there is a trade-off between activity (i.e. second-order rate constant) and specialization. TriA has a large number of promiscuous activities, whilst AtzA is highly specialized, possessing hydrolytic activity only against halides. Here, we have demonstrated that at least one of those promiscuous activities (–SCH_3_ hydrolysis using ametryn) trades-off with atrazine dechlorinase in a similar manner as melamine deaminase activity, with the specificity for ametryn largely influenced again by the identity of the amino acids at positions 331 and 328. Presumptively, this is because ametryn and melamine hydrolysis have similar mechanistic requirements.

Promiscuity is considered a major factor in determining the evolvability of an enzyme [25], and so it may be that TriA is more evolvable than the more specialized AtzA, despite the two enzymes having almost identical specificities for their substrates (1.5–2.0×10^4^ M^−1^.s^−1^; Figs 3 and 4). It seems that the reaction chemistries of these two enzymes determine their potential for further evolution. These findings suggest that activity and specialization need not always trade-off, albeit it is unclear by how much the specificity of TriA for melamine could be increased without loss of its promiscuous activities.

The roles of functional trade-offs and epistasis in constraining the evolutionary pathways of proteins have become increasingly apparent from laboratory-based forced evolution experiments [2], [3], [4]. However, there have been few examples in which these influences have been quantified in a naturally evolved system [5], [19], [26]. Herein, we have demonstrated that, for our model system at least, functional trade-offs and intramolecular epistasis are themselves reflections of chemical requirements for catalysis and not only constrain the availability of evolutionary trajectories but also potentially impact on the further evolvability of these recently evolved enzymes.

## Materials and Methods

### Chemicals

Unless otherwise stated, chemicals were obtained from Sigma (Australia) and were at least 99% pure.

### Bacterial Growth and DNA Manipulation

*Escherichia coli* JM109 (Promega, USA) was used as the host for cloning and expression throughout this work. Bacterial cultures were routinely grown in Luria broth (LB) [27] or at 37°C on LB mixed with agar (15% w/v). LB was also supplemented with chloramphenicol (34 µg mL^−1^) where required. Electrocompetent cells used for the transformation of *E. coli* JM109 with plasmid DNA were obtained from Promega, USA.

Plasmid DNA was prepared using Qiagen’s plasmid minikit (Qiagen, Australia). Site-directed mutagenesis was achieved by overlap extension PCR [28]. The primers used in this process are described in Table S1 and were synthesized by GeneWorks, Australia. For cloning and mutagenesis, amplicons were generated using Phusion high-fidelity DNA polymerase (Finnzymes, Finland). Amplicons were cloned using *Nde*I and *Bam*HI into pCS150 [20], which had been modified to include an extension that encoded an in-frame *N*-terminal hexa-Histidine tag (Material S1). Restriction enzymes, calf intestinal alkaline phosphatase (CIP), and T4 DNA ligase for this cloning were obtained from New England BioLabs, USA.

DNA sequencing of the individual AtzA variants was done at the Micromon DNA Sequencing Facility (Melbourne, Australia) using the vector-specific primers pCS150F and pCS150R (Table S1).

### Protein Purification

AtzA, TriA, and their intermediate variants were produced in *E. coli* JM109 transformed with appropriate pCS150-derived expression plasmids. Bacterial strains were incubated at 28°C in 50 mL LB for 48 h. Cells were harvested by centrifugation at 5,300 rpm at 4°C for 10 min., resuspended in HEPES buffer (pH 8.0) and then lysed using BugBuster (Novagen, Germany) according to the manufacturer’s instructions. Lysates were clarified by centrifugation and the his-tagged enzymes were purified by metal ion affinity chromatography using TALON resin (Clontech, USA) following the manufacturer’s instructions.

Protein purity was assessed by SDS-PAGE (Fig. S1) using NuPAGE Novex 10% Bis-Tris precast gels (Invitrogen, USA) stained with Coomassie Brilliant Blue (Sigma-Aldrich, USA). Protein concentrations were estimated by measuring absorbance at 280 nm using a ND-1000 Nanodrop spectrophotometer (Thermo Fisher Scientific, Australia). The molar extinction coefficient of AtzA was estimated at 53,860 M^−1^.cm^−1^ and that of TriA at 59,360 M^−1^.cm^−1^ using ProtParam [29] (hosted at the ExPASy server: http://www.expasy.org/tools/protparam.html). The molar extinction coefficients of the intermediate variants were estimated by the same method, and yielded values between those of the parent enzymes.

### Enzyme Kinetics

Atrazine and ametryn hydrolyses were monitored by UV-visible spectroscopy at 265 nm as reported elsewhere [12]. Melamine hydrolysis was monitored by measuring the increase in absorbance at 230 nm caused by the accumulation of the hydrolysis product ammeline, a method validated by HPLC analysis using authentic ammeline standards (Material S1). UV-visible spectroscopy was conducted using a SpectraMAX 190 spectrophotometer (Molecular Devices, USA). Enzymes were used at a final concentration of 100 nM in 25 mM MOPS (3-(*N*-Morpholino)-propanesulfonic acid) buffer (pH 6.9) with substrate concentrations in the range of 0 µM to 150 µM at 25°C. For all the enzyme variants tested, the *K*_M_ for both atrazine and ametryn was much higher than 150 µM, so the second order rate constant (*k*_cat_/*K*_M_) was used estimated for all three substrates (atrazine, ametryn and melamine) under the assumption that V_0_≈ *k*_cat_/*K*_M_ [E][S] when [S] << *K*_M_.

### Thermal Stability

The apparent melting temperatures (T_m_^app^) of AtzA, TriA, and selected intermediates were estimated by incubating cell-free extracts for 15 min. at 30°C to 70°C. Residual activities of the enzymes were then determined by UV-visible spectroscopy using atrazine or melamine as substrates.

## Supporting Information

Figure S1 **Purified enzyme variants.** A) SDS-PAGE gel showing purified AtzA variants from the first generation of the AtzA to TriA trajectory. M =  Marker (Precision Plus Protein Standards Dual Color, Bio-Rad); 1 =  AtzA F84L; 2 =  AtzA V92L; 3 =  AtzA E125D, 4 =  AtzA T217I; 5 =  AtzA T219P; 6 =  AtzA I253L; 7 =  AtzA G255W; 8 =  AtzA N328D; 9 =  AtzA S331C. B) SDS-PAGE gel showing purified TriA variants from the TriA to AtzA trajectory. M =  Marker (Precision Plus Protein Standards Dual Color, Bio-Rad); 1 =  TriA L84F; 2 =  TriA L92V; 3 =  TriA D125E, 4 =  TriA I217T; 5 =  TriA P219T; 6 =  TriA L253I; 7 =  TriA W255G; 8 =  TriA D328N; 9 =  TriA C331S; 10 =  TriA L84F-D328N; 11 =  TriA L84F-C331S; 12 =  TriA D328N-C331S; 13 =  TriA L84F-D328N-C331S.
(DOC)

Table S1 **Oligonucleotide primers used in this study.**
(DOC)

Table S2 **Kinetic data for all variants studied.** The identity of the variants, specificity constants (*k*_cat_/*K*_M_) and standard deviations (SD) are shown.
(XLS)

Material S1 **Supporting information.** The full DNA sequence of pCS150 (with in frame his-tag), LC-MS validation of the melamine UV-vis assay and denaturation curves for wild-type AtzA and TriA are shown.
(DOCX)
