# Supplementary material for: Intramolecular Epistasis and the Evolution of a New Enzymatic Function
Source: PLoS One. 2012 Jun 29;7(6):e39822. doi: 10.1371/journal.pone.0039822 (PMC3387218; doi:10.1371/journal.pone.0039822)
Supplement: Figure S1 — Purified enzyme variants. A) SDS-PAGE gel showing purified AtzA variants from the first generation of the AtzA to TriA trajectory. M = Marker (Precision Plus Protein Standards Dual Color, Bio-Rad); 1 = AtzA F84L; 2 = AtzA V92L; 3 = AtzA E125D, 4 = AtzA T217I; 5 = AtzA T219P; 6 = AtzA I253L; 7 = AtzA G255W; 8 = AtzA N328D; 9 = AtzA S331C. B) SDS-PAGE gel showing purified TriA variants from the TriA to AtzA trajectory. M = Marker (Precision Plus Protein Standards Dual Color, Bio-Rad); 1 = TriA L84F; 2 = TriA L92V; 3 = TriA D125E, 4 = TriA I217T; 5 = TriA P219T; 6 = TriA L253I; 7 = TriA W255G; 8 = TriA D328N; 9 = TriA C331S; 10 = TriA L84F-D328N; 11 = TriA L84F-C331S; 12 = TriA D328N-C331S; 13 = TriA L84F-D328N-C331S. (DOC) [file pone.0039822.s001.doc]

**A)**

**
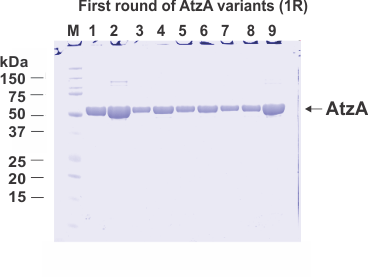
**

**B)**

**
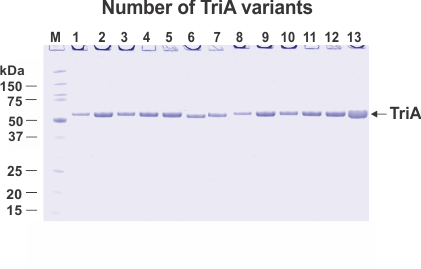
**

**Supp. Fig 1.**

**A) SDS-PAGE gel showing purification of first round (1R) of AtzA variants for AtzA to TriA pathway.** M= Marker (Precision Plus Protein Standards Dual Color, Bio-Rad); 1= AtzA F84L; 2= AtzA V92L; 3= AtzA E125D, 4= AtzA T217I; 5= AtzA T219P; 6= AtzA I253L; 7= AtzA G255W; 8= AtzA N328D; 9= AtzA S331C.

**B) SDS-PAGE gel showing purification of all TriA variants made for TriA to AtzA pathway.** M= Marker (Precision Plus Protein Standards Dual Color, Bio-Rad); 1= TriA L84F; 2= TriA L92V; 3= TriA D125E, 4= TriA I217T; 5= TriA P219T; 6= TriA L253I; 7= TriA W255G; 8= TriA D328N; 9= TriA C331S; 10= TriA L84F-D328N; 11= TriA L84F-C331S; 12= TriA D328N-C331S; 13= TriA L84F-D328N-C331S.
