# Supplementary material for: Intramolecular Epistasis and the Evolution of a New Enzymatic Function
Source: PLoS One. 2012 Jun 29;7(6):e39822. doi: 10.1371/journal.pone.0039822 (PMC3387218; doi:10.1371/journal.pone.0039822)
Supplement: Table S1 — Oligonucleotide primers used in this study. (DOC) [file pone.0039822.s002.doc]

Oligonucleotide primers used by Noor *et al*.:

| **Primer** | **Sequence (5`-3`)** | **Description** |
| --- | --- | --- |
| *atzA*-1F  *atzA*-1R | TCACGGGCGTCAACTCTATGACTGGCTGT  AACAGCCAGTCATAGAGTTGACGCCCGTG | Introduced single mutation for F84L. |
| *atzA*-2F  *atzA*-2R | TGGCTGTTCAACGTTTTGTACCCGGGACA  TGTCCCGGGTACAAAACGTTGAACAGCCA | Introduced single mutation for V92L. |
| *atzA*-3F  *atzA*-3R | CGACGATCAACGACAACGCCGACTCGGCCA  GGCCGAGTCGGCGTTGTCGTTGATCGTCGT | Introduced single mutation for E125D. |
| *atzA*-4F  *atzA*-4R | CGGCCCCTGCCATTACCACGGCGGTGA  CTGTCACCGCCGTGGTAATGGCAGGGG | Introduced single mutation for T217I. |
| *atzA*-5F  *atzA*-5R | CCCTGCCACTACCCCGGCGGTGACAGTTG  CAACTGTCACCGCCGGGGTAGTGGCAGGG | Introduced single mutation for T219P. |
| *atzA*-6F  *atzA*-6R | GATCATGATGAACGACTTCATGGGATGAG  TCATCCCATGAAGTCGTTCATCATGATCC | Introduced single mutation for I253L. |
| *atzA*-7F  *atzA*-7R | GAACGAATTCATTGGATGAGTCCCGCCGA  TCGGCGGGACTCATCCAATGAATTCGTTC | Introduced single mutation for G255W. |
| *atzA*-8F  *atzA*-8R | GGGATCGGTACCGATGACGGGAATAGTAA  TTACTATTCCCGTCATCGGTACCGATCCC | Introduced single mutation for N328D. |
| *atzA*-9F  *atzA*-9R | ATAACGGGAATTGTAATGACTCCGTCAAC  TTGACGGAGTCATTACAATTCCCGTTATC | Introduced single mutation for S331C. |
| *atzA*-10F  *atzA*-10R | CGGCCCCTGCCATTACCCCGGCGGTGACA  TGTCACCGCCGGGGTAATGGCAGGGGCCG | Introduced double mutations for T217I and T219P. |
| *atzA*-11F  *atzA*-11R | TGATGAACGACTTCATTGGATGAGTCCCG  CGGGACTCATCCAATGAAGTCGTTCATCA | Introduced double mutations for I253L and G255W. |
| *atzA*-12F  *atzA*-12R | GGATCGGTACCGATGACGGGAATTGTAATGACTC  GAGTCATTACAATTCCCGTCATCGGTACCGATCC | Introduced double mutations for N328D and S331C. |
| *triA*-1F  *triA*-1R | TCACGGGCGTCAATTCTATGACTGGCTGT  AACAGCCAGTCATAGAATTGACGCCCGTG | Introduced single mutation for L84F. |
| *triA*-2F  *triA*-2R | TGGCTGTTCAACGTTGTGTACCCGGGACA  TGTCCCGGGTACACAACGTTGAACAGCCA | Introduced single mutation for L92V. |
| *triA*-3F  *triA*-3R | CGACGATCAACGAAAACGCCGACTCGGCCA  GGCCGAGTCGGCGTTTTCGTTGATCGTCGT | Introduced single mutation for D125E. |
| *triA*-4F  *triA*-4R | CGGCCCCTGCCACTACCCCGGCGGTGA  CTGTCACCGCCGGGGTAGTGGCAGGGG | Introduced single mutation for I217T. |
| *triA*-5F  *triA*-5R | CCCTGCCATTACCACGGCGGTGACAGTTG  CAACTGTCACCGCCGTGGTAATGGCAGGG | Introduced single mutation for P219T. |
| *triA*-6F  *triA*-6R | GATCATGATGAACGAATTCATTGGATGAG  TCATCCAATGAATTCGTTCATCATGATCC | Introduced single mutation for L253I. |
| *triA*-7F  *triA*-7R | GAACGACTTCATGGGATGAGTCCCGCCGA  TCGGCGGGACTCATCCCATGAAGTCGTTC | Introduced single mutation for W255G. |
| *triA*-8F  *triA*-8R | GGGATCGGTACCGATAACGGGAATTGTAA  TTACAATTCCCGTTATCGGTACCGATCCC | Introduced single mutation for D328N. |
| *triA*-9F  *triA*-9R | ATGACGGGAATAGTAATGACTCCGTCAAC  TTGACGGAGTCATTACTATTCCCGTCATC | Introduced single mutation for C331S. |
| *triA*-10F  *triA*-10R | CGGCCCCTGCCACTACCACGGCGGTGACA  TGTCACCGCCGTGGTAGTGGCAGGGGCCG | Introduced double mutations for I217T and P219T. |
| *triA*-11F  *triA*-11R | TGATGAACGAATTCATGGGATGAGTCCCG  CGGGACTCATCCCATGAATTCGTTCATCA | Introduced double mutations for L253I and W255G. |
| *triA*-12F  *triA*-12R | GGATCGGTACCGATAACGGGAATAGTAATGACTC  GAGTCATTACTATTCCCGTTATCGGTACCGATCC | Introduced double mutations for D328N and C331S. |
| *atzA*-F  *atzA*-R | AGAAGAACATATGCAAACCCTGTCCATCCAGCACGGGACT  TGTTGGTTGGATCCTTAGAGGCTGCGCCAAGC | (5′ - 3′) primers used to amplify full length *atzA* and *triA* mutant genes and in combination with mutagenic primers to introduce point mutations |
