# Supplementary material for: Intramolecular Epistasis and the Evolution of a New Enzymatic Function
Source: PLoS One. 2012 Jun 29;7(6):e39822. doi: 10.1371/journal.pone.0039822 (PMC3387218; doi:10.1371/journal.pone.0039822)
Supplement: Material S1 — Supporting information. The full DNA sequence of pCS150 (with in frame his-tag), LC-MS validation of the melamine UV-vis assay and denaturation curves for wild-type AtzA and TriA are shown. (DOCX) [file pone.0039822.s004.docx]

**Full DNA sequence of the modified pCS150 expression vector with introduced his-tag for protein expression:**

tatgcaaacgctcagcatccagcacggtaccctcgtcacgatggatcagtaccgcagagtccttggggatagctgggttcacgtgcaggatggacggatcgtcgcgctcggagtgcacgccgagtcggtgcctccgccagcggatcgggtgatcgatgcacgcggcaaggtcgtgttacccggtttcatcaatgcccacacccatgtgaaccagatcctcctgcgcggagggccctcgcacgggcgtcaattctatgactggctgttcaacgttgtgtatccgggacaaaaggcgatgagaccggaggacgtagcggtggcggtgaggttgtattgtgcggaagctgtgcgcagcgggattacgacgatcaacgaaaacgccgattcggccatctacccaggcaacatcgaggccgcgatggcggtctatggtgaggtgggtgtgagggtcgtctacgcccgcatgttctttgatcggatggacgggcgcattcaagggtatgtggacgccttgaaggctcgctctccccaagtcgaactgtgctcgatcatggaggaaacggctgtggccaaagatcggatcacagccctgtcagatcagtatcatggcacggcaggaggtcgtatatcagtttggcccgctcctgccactaccacggcggtgacagttgaaggaatgcgatgggcacaagccttcgcccgtgatcgggcggtaatgtggacgcttcacatggcggagagcgatcatgatgagcggattcatgggatgagtcccgccgagtacatggagtgttacggactcttggatgagcgtctgcaggtcgcgcattgcgtgtactttgaccggaaggatgttcggctgctgcaccgccacaatgtgaaggtcgcgtcgcaggttgtgagcaatgcctacctcggctcaggggtggcccccgtgccagagatggtggagcgcggcatggccgtgggcattggaacagataacgggaatagtaatgactccgtaaacatgatcggagacatgaagtttatggcccatattcaccgcgcggtgcatcgggatgcggacgtgctgaccccagagaagattcttgaaatggcgacgatcgatggggcgcgttcgttgggaatggaccacgagattggttccatcgaaaccggcaagcgcgcggaccttatcctgcttgacctgcgtcaccctcagacgactcctcaccatcatttggcggccacgatcgtgtttcaggcttacggcaatgaggtggacactgtcctgattgacggaaacgttgtgatggagaaccgccgcttgagctttcttccccctgaacgtgagttggcgttccttgaggaagcgcagagccgcgccacagctattttgcagcgggcgaacatggtggctaacccagcttggcgcagcctctaaggatccaagcctatgcctacagcatccagggtgacggtgccgaggatgacgatgagcgcattgttagatttcatacacggtgcctgactgcgttagcaatttaactgtgataaactaccgcattaaagcttatcgatgataagctgtcaaacatgagaattacaacttatatcgtatggggctgacttcaggtgctacatttgaagagataaattgcactgaaatctagaaatattttatctgattaataagatgatcttcttgagatcgttttggtctgcgcgtaatctcttgctctgaaaacgaaaaaaccgccttgcagggcggtttttcgaaggttctctgagctaccaactctttgaaccgaggtaactggcttggaggagcgcagtcaccaaaacttgtcctttcagtttagccttaaccggcgcatgacttcaagactaactcctctaaatcaattaccagtggctgctgccagtggtgcttttgcatgtctttccgggttggactcaagacgatagttaccggataaggcgcagcggtcggactgaacggggggttcgtgcatacagtccagcttggagcgaactgcctacccggaactgagtgtcaggcgtggaatgagacaaacgcggccataacagcggaatgacaccggtaaaccgaaaggcaggaacaggagagcgcacgagggagccgccagggggaaacgcctggtatctttatagtcctgtcgggtttcgccaccactgatttgagcgtcagatttcgtgatgcttgtcaggggggcggagcctatggaaaaacggctttgccgcggccctctcacttccctgttaagtatcttcctggcatcttccaggaaatctccgccccgttcgtaagccatttccgctcgccgcagtcgaacgaccgagcgtagcgagtcagtgagcgaggaagcggaatatatcctgtatcacatattctgctgacgcaccggtgcagccttttttctcctgccacatgaagcacttcactgacaccctcatcagtgccaacatagtaagccagtatacactccgctagcgctgatgtccggcggtgcttttgccgttacgcaccaccccgtcagtagctgaacaggagggacagctgatagaaacagaagccactggagcacctcaaaaacaccatcatacactaaatcagtaagttggcagcatcacccgacgcactttgcgccgaataaatacctgtgacggaagatcacttcgcagaataaataaatcctggtgtccctgttgataccgggaagccctgggccaacttttggcgaaaatgagacgttgatcggcacgtaagaggttccaactttcaccataatgaaataagatcactaccgggcgtattttttgagttatcgagattttcaggagctaaggaagctaaaatggagaaaaaaatcactggatataccaccgttgatatatcccaatggcatcgtaaagaacattttgaggcatttcagtcagttgctcaatgtacctataaccagaccgttcagctggatattacggcctttttaaagaccgtaaagaaaaataagcacaagttttatccggcctttattcacattcttgcccgcctgatgaatgctcatccggaattccgtatggcaatgaaagacggtgagctggtgatatgggatagtgttcacccttgttacaccgttttccatgagcaaactgaaacgttttcatcgctctggagtgaataccacgacgatttccggcagtttctacacatatattcgcaagatgtggcgtgttacggtgaaaacctggcctatttccctaaagggtttattgagaatatgtttttcgtctcagccaatccctgggtgagtttcaccagttttgatttaaacgtggccaatatggacaacttcttcgcccccgttttcaccatgggcaaatattatacgcaaggcgacaaggtgctgatgccgctggcgattcaggttcatcatgccgtctgtgatggcttccatgtcggcagaatgcttaatgaattacaacagtactgcgatgagtggcagggcggggcgtaatttttttaaggcagttattggtgcccttaaacgcctggtgctacgcctgaataagtgataataagcggatgaatggcagaaattcgaaagcaaattcgacccggtcgtcggttcagggcagggtcgttaaatagccgcttatgtctattgctggtttaccggtttattgactaccggaagcagtgtgaccgtgtgcttctcaaatgcctgaggccagtttgctcaggctctccccgtggaggtaataattgacgatatgatcatttattctgcctcccagagcctgataaaaacggttagcgcttcgttaatacagatgtaggtgttccacagggtagccagcagcatcctgcgatgcagatccggaacataatggtgcagggcgcttgtttcggcgtgggtatggtggcaggccccgtggccgggggactgttgggcgctgccggcacctgtcctacgagttgcatgataaagaagacagtcataagtgcggcgacgatagtcatgccccgcgcccaccggaaggagctaccggacagcggtgcggactgttgtaactcagaataagaaatgaggccgctcatggcgttgactctcagtcatagtatcgtggtatcaccggttggttccactctctgttgcgggcaacttcagcagcacgtaggggacttccgcgtttccagactttacgaaacacggaaaccgaagaccattcatgttgttgctcaggtcgcagacgttttgcagcagcagtcgcttcacgttcgctcgcgtatcggtgattcattctgctaaccagtaaggcaaccccgccagcctagccgggtcctcaacgacaggagcacgatcatgcgcacccgtggccaggacccaacgctgcccggggaacttcttgagcgcggccacagcagccttgatcatgaaggcgagcatggtgaccttgacgccgctcttttcgttctctttgttgaactgcacgcgaaaggcttccaggtcggtgatgtccgcgtcgtcgtggttggtgacgtgcgggatgaccacccagttgcggtgcaggtttttcgatggcataatatctgcgttgcgacgtgtaacacactattggagacatca

**Method for HPLC analysis of melamine hydrolysis:**

HPLC assay conducted using an Agilent Technologies 1200 series with a Zorbax Eclipse XDB-C18 (2.1 × 30mm, 3.5 µm) rapid resolution column. Briefly, 5 µl of sample was injected onto the column at a flow rate 1 mL.min^-1^ using an isocratic eluent (7% (v/v) acetonitrile, 4.7 mM octancesulfonate with a pH adjusted to 2.5 by addition of phosphoric acid). The run time was 1.2 min with melamine and ammeline (4,6-Diamino-2-hydroxy-1,3,5-triazine) peaks eluted at 0.52 min and 0.68 min, respectively. Standard curves were produced using ammeline concentrations 0-1600 µM. Enzyme assays were conducted using 100 nM TriA, and were in good agreement with the UV-vis assay.

**Melting curves for AtzA and TriA**

**AtzA:**

T_m_^app^ = 54.0 ^o^C

**TriA:**

T_m_^app^ = 44.1 ^o^C
